# Transcriptome Analysis Reveals an Eicosapentaenoic Acid Accumulation Mechanism in a *Schizochytrium* sp. Mutant

**DOI:** 10.1128/spectrum.00130-23

**Published:** 2023-04-24

**Authors:** Ying Ou, Yaqi Li, Shoushuai Feng, Qiong Wang, Hailin Yang

**Affiliations:** a Key Laboratory of Industrial Biotechnology, Ministry of Education, School of Biotechnology, Jiangnan University, WuXi, Jiangsu Province, People’s Republic of China; b Department of Clinical Laboratory, The Affiliated Wuxi People’s Hospital of Nanjing Medical University, People’s Republic of China; University of Minnesota Twin Cities

**Keywords:** ARTP-DES mutagenesis, EPA, *Schizochytrium*, sethoxydim, transcriptomic, triclosan-2, 2′-bipyridine

## Abstract

Eicosapentaenoic acid (EPA) is an omega-3 long-chain polyunsaturated fatty acid (PUFA) essential for human health. *Schizochytrium* is a marine eukaryote that has been widely utilized for the synthesis of PUFAs. The current low potency and performance of EPA production by fermentation of *Schizochytrium* spp. limits its prospect in commercial production of EPA. Since the synthesis pathway of EPA in *Schizochytrium* spp. is still unclear, mutagenesis combined with efficient screening methods are still desirable. In this study, a novel screening strategy was developed based on a two-step progressive mutagenesis method based on atmospheric and room temperature plasma (ARTP) and diethyl sulfate (DES) after multiple stresses (sethoxydim, triclosan and 2,2′-bipyridine) compound screening. Finally, the mutant strain DBT-64 with increased lipid (1.57-fold, 31.71 g/L) and EPA (5.64-fold, 1.86 g/L) production was screened from wild-type (W) strains; the docosahexaenoic acid (DHA) content of mutant DBT-64 (M) was 11.41% lower than that of wild-type strains. Comparative transcriptomic analysis showed that the expression of genes related to the polyketide synthase, fatty acid prolongation, and triglyceride synthesis pathways was significantly upregulated in the mutant strain, while the expression of genes involved in the β-oxidation pathway and fatty acid degradation pathway was downregulated in favor of EPA biosynthesis in *Schizochytrium*. This study provides an effective strain improvement method to enhance EPA accumulation in *Schizochytrium* spp.

**IMPORTANCE** *Schizochytrium*, a marine eukaryotic microorganism, has emerged as a candidate for the commercial production of PUFAs. EPA is an omega-3 PUFA with preventive and therapeutic effects against cardiovascular diseases, schizophrenia, and other disorders. Currently, the low potency and performance of EPA production by *Schizochytrium* spp. limits its commercialization. In this study, we performed two-step progressive mutagenesis based on ARTP and DES and screened multiple stresses (sethoxydim, triclosan, and 2,2′-bipyridine) to obtain the EPA-high-yielding *Schizochytrium* mutant. In addition, high expression of the polyketide synthase pathway, fatty acid elongation pathway, and triglyceride synthesis pathway in the mutants was confirmed by transcriptomic analysis. Therefore, the multistress screening platform established in this study is important for breeding EPA-producing *Schizochytrium* spp. and provides valuable information for regulating the proportion of EPA in microalgal lipids by means of genetic engineering.

## INTRODUCTION

Eicosapentaenoic acid (EPA) is an omega-3-polyunsaturated fatty acid (PUFA) that is important for the human body. Due to its significant physiological roles in the prevention and treatment of cardiovascular diseases (1), schizophrenia and depression (2), and inflammation and cancer (3, 4), EPA has received extensive attention. Currently, fish oil is the most important dietary source of EPA for the global market (5), but the supply of high-quality fish oil has declined considerably due to factors such as the increasing depletion of marine resources and environmental pollution. Marine microalgae are the primary producers of PUFAs. The safe and controlled production process and the absence of arable land requirements have made marine microalgae widely appreciated as an alternative source for the production of PUFAs. Common EPA-producing microalgae include Phaeodactylum tricornutum (6), Nitzschia laevis (7), and *Nannochloropsis* spp. (8). Due to their weak ultimate production in the fermentation process, the development of the industrial production of EPA has encountered a bottleneck. Therefore, researchers have been working to select and breed efficient EPA producers.

*Schizochytrium* is a heterotrophic marine fungus with the advantages of high PUFA content, fast growth rate, and low fermentation cost, and it has become a candidate for the commercial production of PUFAs. There are two fatty acid synthesis pathways in *Schizochytrium* spp.; the fatty acid synthase (FAS) pathway mainly synthesizes short-chain saturated fatty acids, and the polyketide synthase (PKS) pathway synthesizes PUFAs (9). In recent years, the lipid content and docosahexaenoic acid (DHA) production in *Schizochytrium* have been enhanced by mutagenesis, genetic engineering, and optimization of the fermentation process (10–12). However, *Schizochytrium*, as a nonmodel strain, faces problems such as few gene expression elements and unclear lipid metabolism pathways during genetic engineering modification. Therefore, improving the strain by physical or chemical mutagenesis is one of the most common methods to obtain a *Schizochytrium* sp. with desirable characteristics (11).

Atmospheric and room temperature plasma (ARTP) mutagenesis technology has been widely used for the breeding of marine microorganisms, including *Schizochytrium* spp., because of its high positive efficiency and numerous phenotypic mutations (13). Studies have shown that mutagenesis induced by compound rationalization factors is superior to single-factor mutagenesis (14–17). An effective screening method after mutagenesis is a critical step to obtain mutant strains with desirable characteristics (18). Traditional screening methods such as phenotypic screening and morphological screening are tedious and time-consuming (19). Therefore, more efficient screening methods are essential for the development of lipid-producing microbial strains capable of yielding substantial quantities of PUFAs. The acetyl-coenzyme A (CoA) production pathway, NADPH production pathway, and fatty acid synthase are important metabolic modules for achieving efficient accumulation of PUFAs in marine microorganisms (20), and inhibitors targeting several modules have been widely used to screen mutant strains. Malonic acid (MA) competitively inhibits succinate dehydrogenase in the tricarboxylic acid cycle (TCA), resulting in a large accumulation of citric acid in mitochondria and providing more precursors for fatty acid synthesis (21). Acetyl-CoA carboxylase (ACACA) is the first step in the fatty acid biosynthetic pathway, catalyzing the carboxylation of acetyl-CoA to malonyl-CoA (22). Upregulation of ACACA activity has been shown to significantly increase fatty acid production in several oleaginous microbes (23–25). In addition, screening strategies based on ACACA inhibitors (clethodim) have been successfully applied to screen for high-DHA-yield mutants of *Schizochytrium* (13). Triclosan has been widely used as an enoyl reductase (ER) inhibitor to regulate fatty acid synthesis (26). By adding triclosan to inhibit the conversion of butenyl-ACP to butyryl-ACP, the yield of crotonic acid was improved by Liu et al. (27). Moreover, the addition of triclosan significantly increased the DHA content and lipid content of *Aurantiochytrium* (28). PUFAs play an important role in resistance to environmental stress. Kim et al. concluded that DHA and EPA, as antioxidants in biological organisms, could avoid the peroxidation caused by reactive oxygen species (ROS) (29). Therefore, strains with higher antioxidant capacity screened by mutagenesis may have higher DHA or EPA production capacity.

In this study, a high-throughput screening based on Nile Red staining was performed on the strain *Schizochytrium* ATCC 20888 using a combination of ARTP mutagenesis and sethoxydim pressure to obtain strains with high lipid content. Subsequently, the strain with high lipid content was subjected to diethyl sulfate (DES) mutagenesis and triclosan-2,2′-bipyridine compound screening, and finally a mutant strain with significantly elevated lipid content and EPA content was obtained. In addition, the mutant and wild-type strains were compared by sequence transcriptome analysis to investigate the mechanism associated with the increased EPA content of the mutant strain. The screening strategy developed in this paper provides an effective means for the mutagenesis and breeding of *Schizochytrium* to produce EPA, and uncovering the relevant mechanisms will provide valuable regulatory targets for designing *Schizochytrium* with higher EPA titer in the future.

## RESULTS

### Screening of high-lipid-content mutants by atmospheric and room temperature plasma (ARTP) mutagenesis based on sethoxydim.

**FIG 1:**
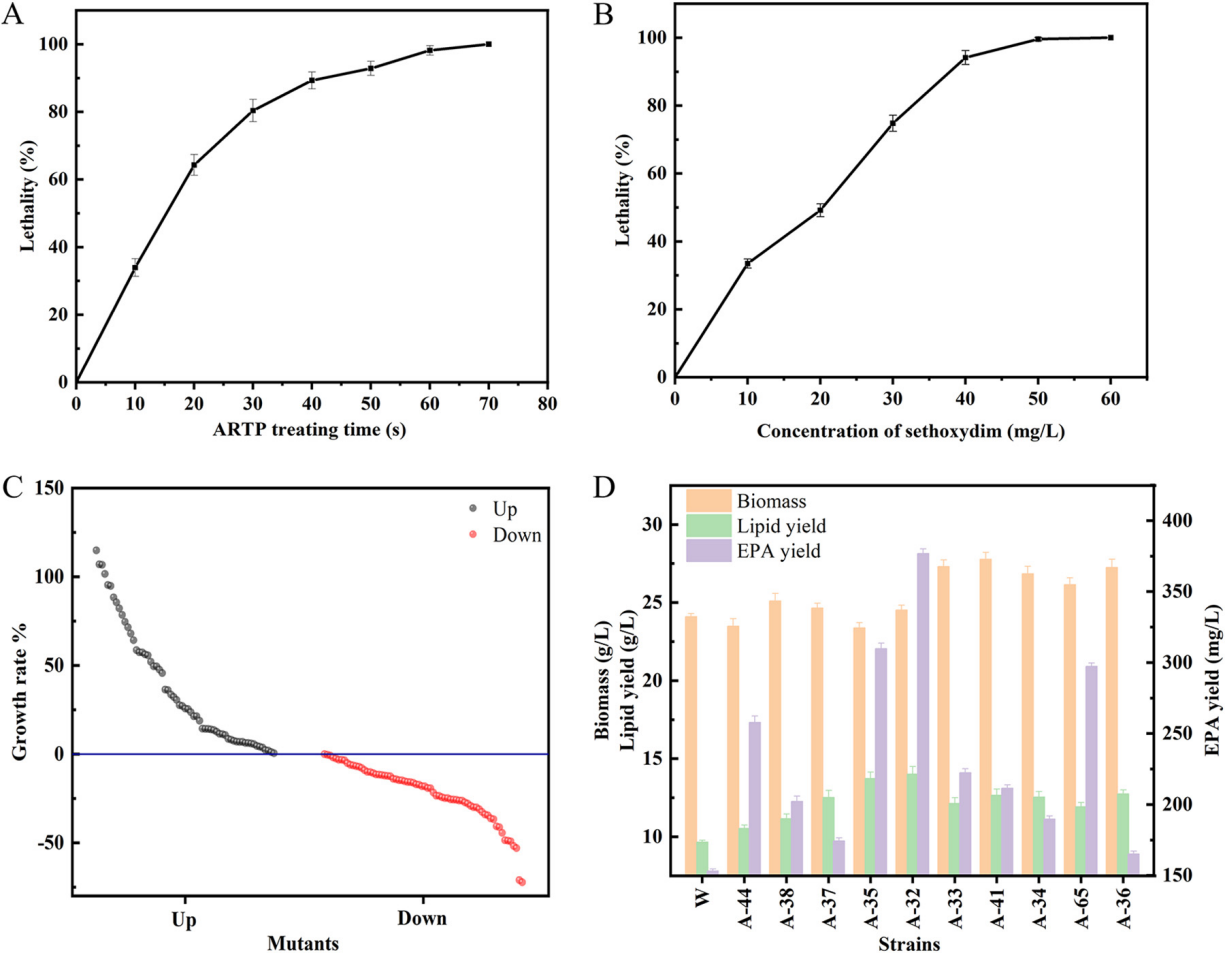
Sensitivity of the *Schizochytrium* ATCC 20888 strain to ARTP and sethoxydim and the mutant after screening. (A) ARTP treatment time. (B) Sensitivity of *Schizochytrium* ATCC 20888 to sethoxydim. (C) Screening of mutant library. (D) Biomass, EPA yield, and lipid content of wild-type strains and candidate mutants. The error bars represent the standard deviation based on three independent measurements.

The lethality rate was selected as the parameter, and the suspension of *Schizochytrium* ATCC 20888 was treated with ARTP. As shown in Fig. 1A, the lethality rate expanded dramatically with increasing treatment time, indicating that *Schizochytrium* cells were extremely sensitive to ARTP treatment time. The lethality rate reached 92.86% at the treatment time of 50 s. Subsequently, the increase in the lethality rate slowed, which was regarded as a sign of activation of internal cellular repair mechanisms. According to reports, a lethality range of 90% to 99% is more effective in mutagenesis, and a lower lethality rate favors the generation of positive mutants (30). Therefore, the treatment time of ARTP mutagenesis was determined to be 50 s in subsequent experiments.

Screening plates containing various concentrations of sethoxydim were prepared to determine the susceptibility of *Schizochytrium*. The lethality rate increased with increasing concentrations of sethoxydim. When the concentration of sethoxydim was greater than 40 mg/L, the lethality rates of *Schizochytrium* reached 94.46% (Fig. 1B). Therefore, the screening concentration of sethoxydim was 40 mg/L.

A mutagenesis library with approximately 5,000 clones was constructed using the optimized parameters for mutagenesis treatment described in Materials and Methods. During the Nile Red staining procedure, the various densities of mutant cells due to differences in inoculum and growth conditions directly affected the choice of high or low lipid content. Therefore, we employed the ratio of fluorescence intensity and optical density at 595 nm (FI/OD_595_) to express the amount of lipid per unit cell density of *Schizochytrium*. High-throughput screening of a library of mutants of *Schizochytrium* was performed using a multifunctional microplate reader. In order to further improve the accuracy of the screening, the top 5 mutants with FI/OD_595_ values in each of the two 96-well plates were initially screened, and then the first round of screening was repeated (Fig. S1). The FI/OD_595_ values of wild-type strains (W) were used as a baseline to classify mutants with increased and decreased lipid levels (Fig. 1C). In the increased lipid content group, four mutant strains showed a substantial increase in FI/OD_595_ values by more than 100% compared to the W, while in the decreased lipid group, most of the mutants had FI/OD_595_ values concentrated at about 75% of the W, and the mutant with the lowest FI/OD_595_ value was only 27.73% of the W.

To verify that the screened mutant strains had high lipid content, 10 mutants with high FI/OD_595_ values were selected for shake flask culture to evaluate lipid content. The results are shown in Fig. 1D. The lipid content of the mutant strain was significantly higher than that of the wild type (10.81% to 46.54%). The lipid content of mutant A-32 reached 57.15% of the biomass and was therefore selected as the starting strain for the next round of mutagenesis.

### Screening of high-EPA-yield mutants by DES mutagenesis based on 2,2′-bipyridine and triclosan.

We used DES to mutagenize S*chizochytrium* A-32 for diverse times, and the cell lethality curve is shown in Fig. S2C in the supplemental material. Cell lethality increased rapidly to 93.29% within 15 min and then leveled off. Therefore, a DES mutagenesis time of 15 min was selected. After DES mutagenesis, high-EPA-producing strains were isolated utilizing a screening plate involving a mixed screening agent1 (2,2′-bipyridine and triclosan at a concentration ratio of 5:3) at 10 mg/L (Fig. S2D).

The DES mutagenesis experiment was carried out according to the above optimized parameters, and cells were cultured on a screening plate containing 10 mg/L of 2,2′-bipyridine and triclosan at a mixed concentration of 10 mg/L for 4 days. A total of 80 mutants with relatively large colonies were selected for evaluation of EPA yield by shake flask culture, and the results are shown in Fig. S3. Among them, DBT-9, DBT-11, DBT-14, and DBT-64 mutants had the highest relative EPA yield.

**FIG 2:**
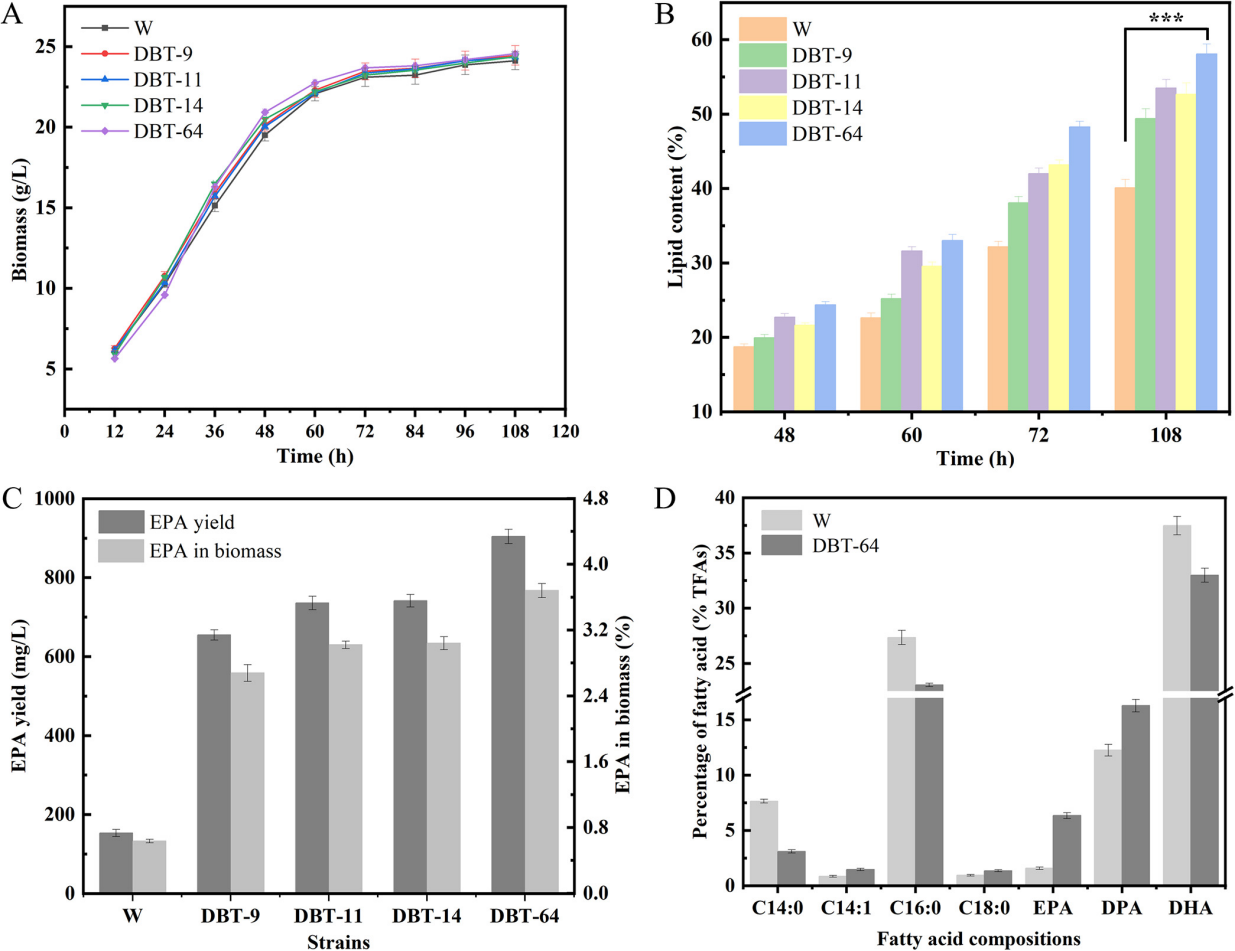
Biomass, lipid content, EPA yield, EPA content (percent biomass), and fatty acid compositions of the wild-type (W) and mutant strains in shake-flask culture. (A) Biomass accumulation. (B) Lipid content. (C) EPA yield and EPA content (percent biomass). (D) Fatty acid compositions. All data were collected from three independent experiments. Error bars represent the standard deviation of three biological replicates. ***, *P* < 0.001.

The mutants DBT-9, DBT-11, DBT-14, and DBT-64 were fermented in shake flasks with the wild-type strain as the control, and their biomass, fatty acid content, and EPA yield were determined. As shown in Fig. 2A, the growth tendency of the 4 mutants was identical to that of the wild type, the biomass increased with the increase of fermentation time, and the biomass at the end of fermentation was slightly higher than that of the wild type. The lipid content of all tested mutants was significantly higher than that of the wild type at 72 h and 108 h, and the lipid accumulation rate of mutant DBT-64 was as high as 58.07%, which was 44.85% higher than that of wild-type strain (Fig. 2B).

Comparative analysis of the EPA yields revealed that all tested mutants were significantly higher than the wild-type strain (Fig. 2C). Among them, the mutant DBT-64 had the highest EPA production and EPA percentage in biomass, reaching 904.21 mg/L and 3.68% (EPA/biomass percentage), respectively, which were 5.90 times and 5.80 times higher than those of the wild-type strain. The proportion of EPA in total fatty acids in DBT-64 was much higher than that of the wild type (6.34%, which is 4.01 times that of the wild type) (Fig. 2D). In addition, the C_14:0_ and C_16:0_ contents of DBT-64 decreased from 7.65% to 3.10% and 27.35% to 23.05%, respectively. Interestingly, the DHA content of DBT-64 was only 32.99%, which decreased by 11.96% compared with the wild type, but the DPA content increased from 12.26% to 16.29%. The results suggest that the mutation resulted in the conversion of saturated fatty acids (SFAs) to PUFAs, reflecting a potential mutation in the gene for fatty acid carbon chain elongation and unsaturation. The mutant DBT-64 was passaged multiple times to test its genetic stability, and the results demonstrated that the mutant was genetically stable (Table S2) and could be used for further studies.

### Comparison of the morphological characteristics and lipid distribution between the wild type and mutant DBT-64.

**FIG 3:**
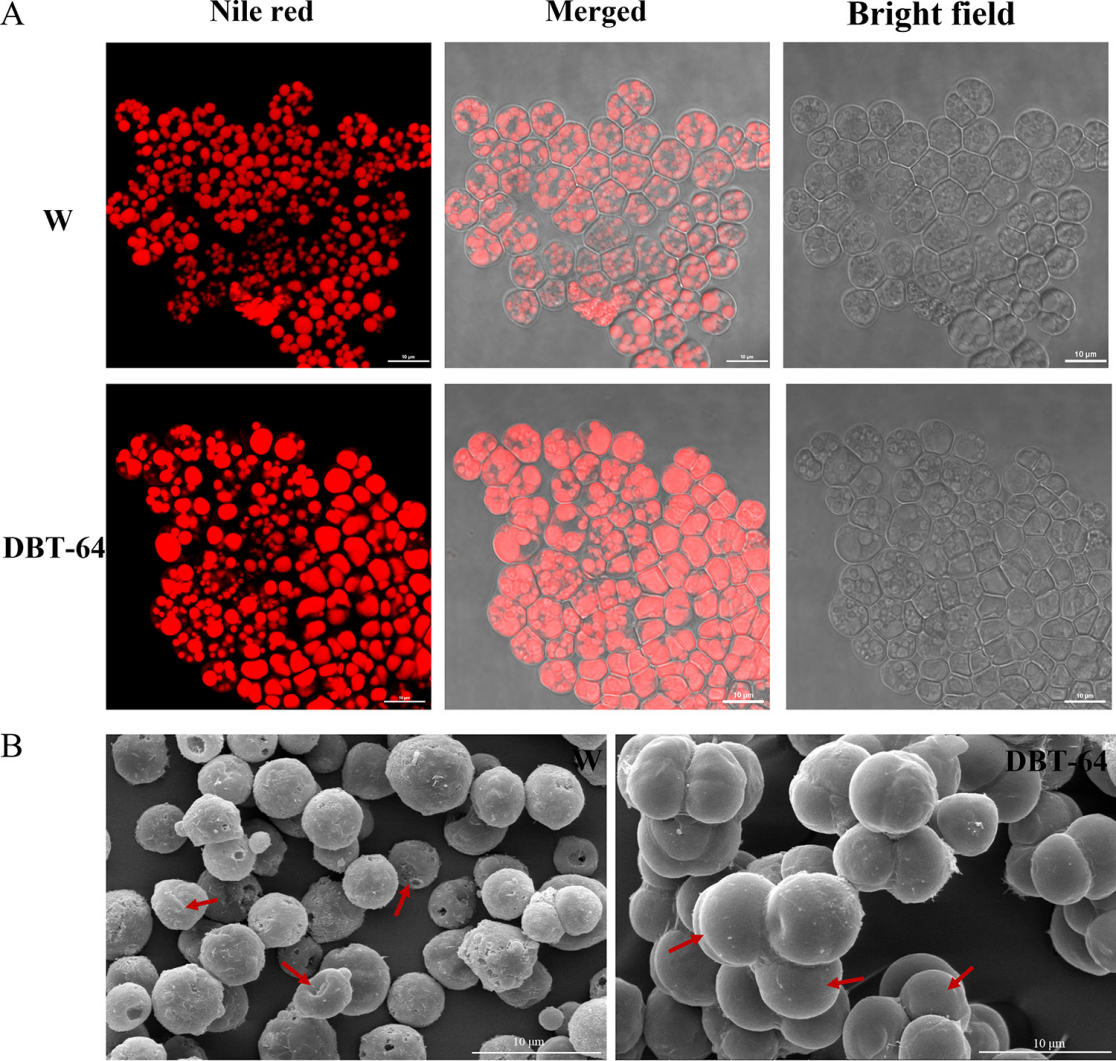
Cell morphological characteristics of the wild-type strain (W) and mutant DBT-64 were analyzed. (A) Morphology of cells stained with Nile Red under LSCM. (B) Morphology of cells at 108 h under SEM.

The mutant DBT-64 and the wild-type strain were cultured for 108 h and stained with Nile Red, and the distribution of neutral lipids in the cells was observed using a laser scanning confocal microscope. As a relatively stable substance, neutral lipids are stored in large quantities in the cytoplasmic liposomes of oleaginous microorganisms. As shown in Fig. 3A, the liposomes in the mutant strain DBT-64 are full in shape, and a considerable number of single liposomes fill the entire cell, while the liposomes in the wild-type strain are small and discrete, and their proportion in the cell and fluorescence strength are much lower than those of DBT-64. Scanning electron microscopy (SEM) images showed obvious differences in cell division between DBT-64 and wild-type strains (Fig. 3B). Most of the cells in the wild-type strains are small, with a wrinkled surface and invagination phenomenon, while DBT-64 cells are mainly divided into vegetative cells with a large volume and smooth surface. Overall, it is evident that the mutant strain DBT-64 has larger cells, larger lipid droplets, and stronger fluorescence intensity. Geng et al. found that recombinant *Schizochytrium* producing high levels of PUFAs had larger cell clusters than wild-type strains (31). The size of the cell is associated with the structure of the cell membrane, which is primarily determined by the overall fatty acids (mainly PUFAs) (32). A high content of PUFAs can increase the comparative fluidity of cell membranes, thereby enhancing the resistance of cells to external environmental pressure (33).

### Production performance evaluation of mutant DBT-64 in a 7.5-L fermenter.

To investigate the growth and production performance of mutant DBT-64, a fed-batch process was performed in a 7.5-L fermenter with the glucose concentration maintained at 20 to 50 g/L. The biomass dry weight, lipid, DHA, DPA, and EPA yields of the *Schizochytrium* wild-type strain and DBT-64 in a 7.5-L fermenter are displayed in Table 1. At the end of fed-batch fermentation, the biomass of the mutant reached 53.59 g/L, which was 8.44% higher than that of the wild-type strain. The lipid content of DBT-64 was considerably higher than that of the W (59.17% and 40.75%, respectively, with an increase of 45.20%), and the total lipid yield was about 31.71 g/L. The EPA yield of DBT-64 was 1.86 g/L, which was 4.64 times higher than that of the wild-type strain, but the DHA content (33.30% of the total fatty acids; the final yield was 10.56 g/L) was lower than that of the wild-type strain (37.59%).

**TABLE 1:** Lipid profile in wild-type and mutant DBT-64 strains at the endpoint fed-batch fermentation

Strain	Biomass (g/L)	Lipid (g/L)	DHA (g/L)	DPA (g/L)	EPA (g/L)
W	49.42 ± 3.86	20.14 ± 2.13	7.57 ± 0.39	2.39 ± 0.14	0.33 ± 0.02
DBT-64	53.59 ± 3.05	31.71 ± 2.65	10.56 ± 0.41	5.53 ± 0.20	1.86 ± 0.14

### Transcriptomic analysis.

To gain a comprehensive understanding of the molecular mechanisms underlying EPA production, comparative transcriptional studies were performed between the W and mutant DBT-64 (M). Samples were collected at 24, 48, and 72 h of fermentation. RNA sequencing of the RNA library using Illumina technology showed 42.53 ± 0.28 million (M) clean reads, of which the GC content and Q30 (the percentage of bases with quality *N*_30_ in clean reads) were 57.65 ± 0.35% and 91.41 ± 0.39%, respectively. After *de novo* assembly of clean reads utilizing Trinity, a total of 23,326 unigenes were generated with a mean length of 1,925 and *N*_50_ value of 2,956 bp, implying that 50% of the assembled reads were incorporated into transcripts longer than 2,569 bp. The summary statistics of the *de novo* assembly and length distribution are presented in Tables S3 and S4 and Fig. S4, respectively, with a total of 4,588 unigene fragments above 3,000 nucleotides (nt) in length.

**FIG 4:**
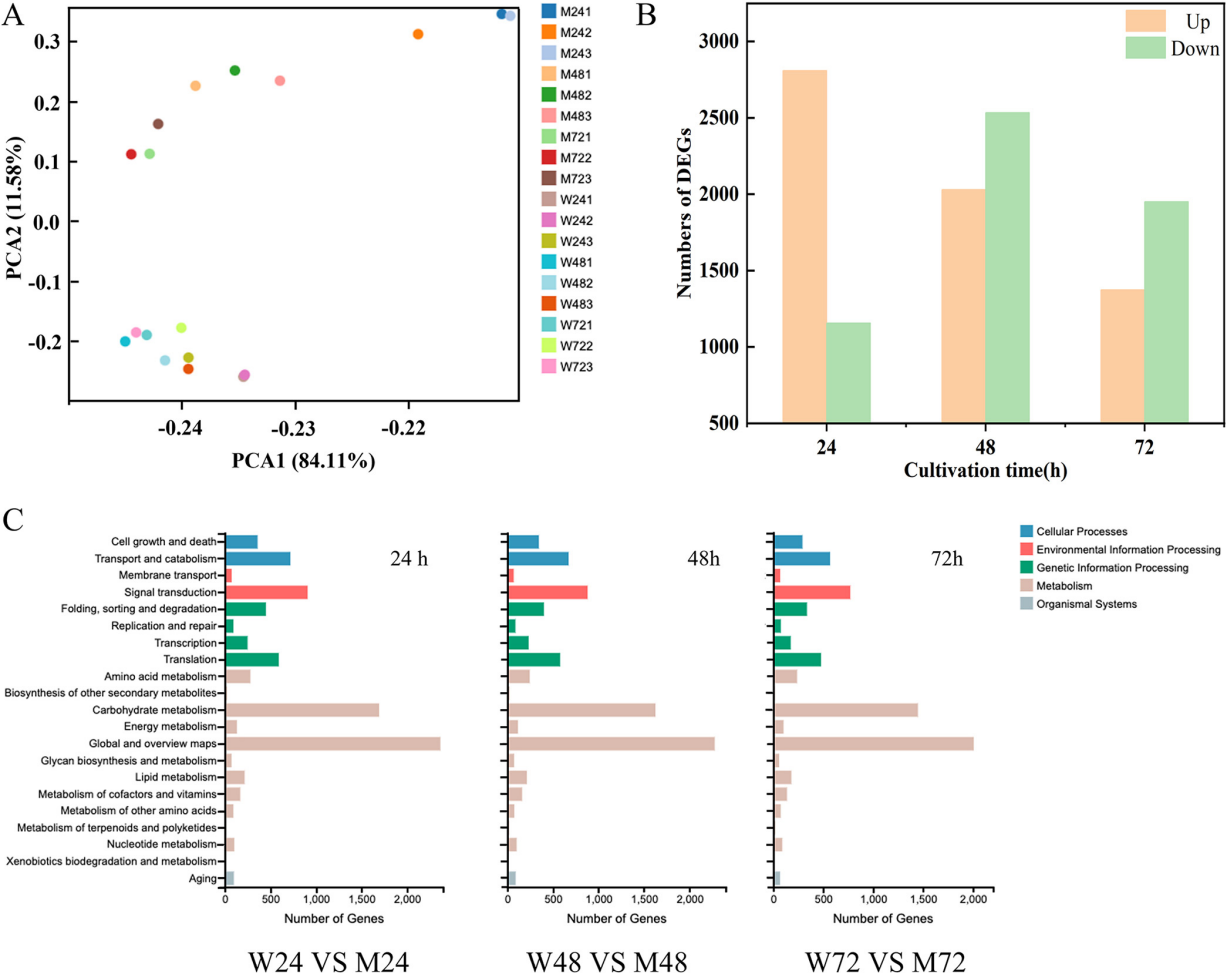
Overview of transcriptome analysis during fermentation with wild-type strains and mutant DBT-64. (A) PCA plot showing clustering between transcriptome samples. (B) The number of DEGs of upregulated and downregulated genes in the fermentation stage between mutant and wild-type strains. (C) KEGG pathway classification analysis of all DEGs. The left side represents the differentially expressed genes between the mutant and wild-type strains at 24 h of fermentation. The middle represents the differentially expressed genes between the mutant and wild-type strains at 48 h of fermentation. The right side represents the differentially expressed genes between the mutant and wild-type strains at 72 h of fermentation. W, wild-type strains; M, mutant DBT-64. Three biological replicates were carried out.

Principal-component analysis (PCA) was performed on all expressed genes in the sample, based on the number of fragments per kilobase per million fragments of transcripts (FPKM). The distances between the clusters of the mutant DBT-64 (M) and wild-type (W) groups of samples were larger at each time point (Fig. 4A), indicating good discrimination between the two groups of samples. Genes with an adjusted *P* value (*P*-adjust) of <0.05 and a log_2_ fold change (FC) of ≥1 were selected as differentially expressed genes (DEGs) for further analysis. Taking the wild type at the same time point as the control, it was found that 2,810, 2,030, and 1,373 genes were upregulated, and 1,156, 2,534, and 1,951 genes were downregulated in the M samples at 24 h, 48 h, and 72 h, respectively (Fig. 4B). To further analyze the molecular mechanisms of EPA synthesis, these genes were classified into 21 major cellular and metabolic processes according to KEGG annotation (as shown in Fig. 4C). These major metabolic pathways are correlated with fatty acid metabolism, carbohydrate metabolism, biosynthesis and translation, and biosynthesis of secondary metabolites. The pathways involved in fatty acid metabolism by according to KEGG include fatty acid metabolism, glycerophospholipid metabolism, triglyceride metabolism, fatty acid biosynthesis, fatty acid elongation, and secondary metabolite biosynthesis, which may play important roles in PUFA biosynthesis.

### Global transcriptional analysis of central carbon metabolism and lipid-related pathways.

**FIG 5:**
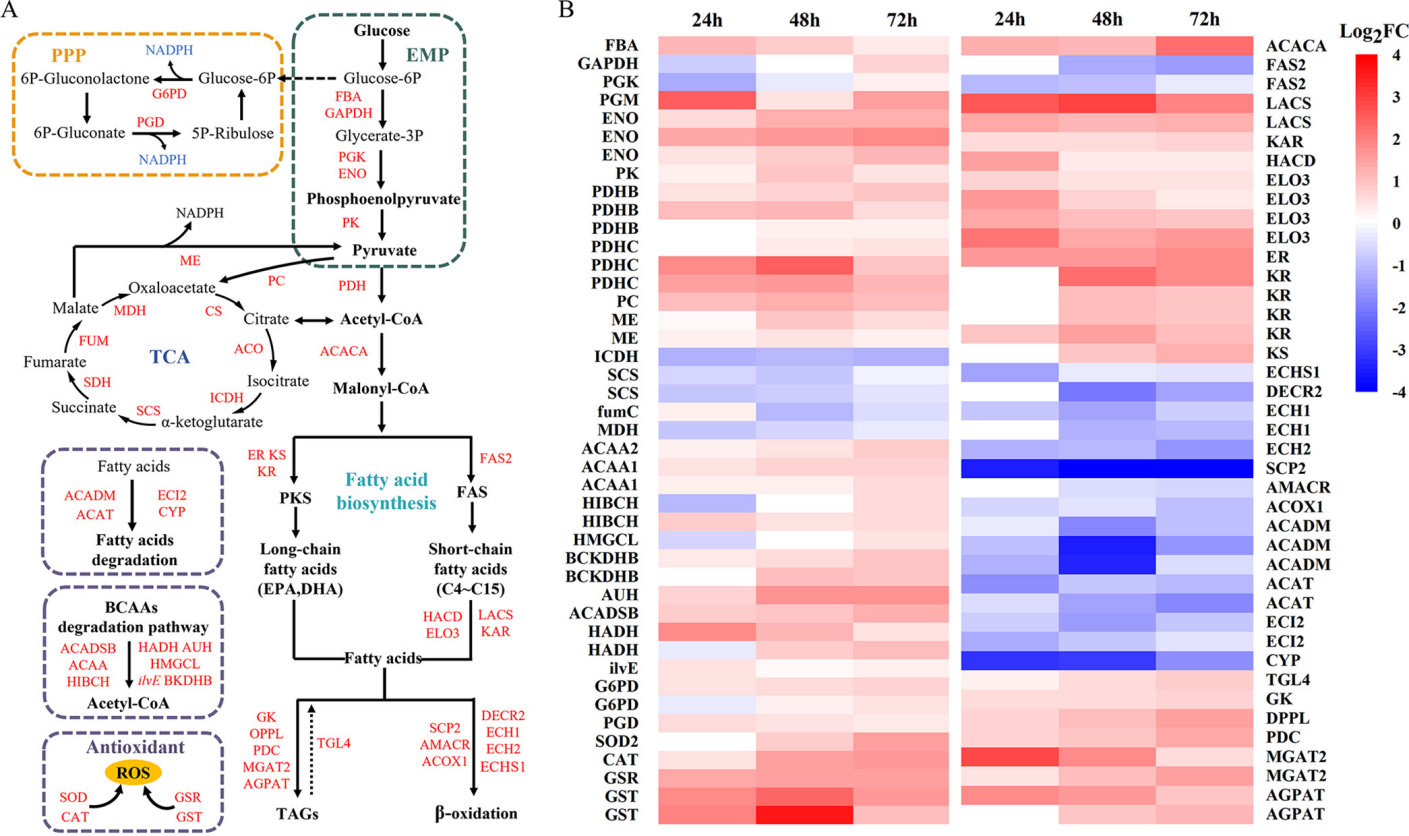
Overview of transcriptional reprogramming of carbon metabolism and lipid-associated pathways in *Schizochytrium*. (A) Schematic map of metabolic pathways on lipid accumulation. (B) Transcriptional differential heat map of log_2_ (fold change) between the wild-type strain and mutant DBT-64 at 24 h, 48 h, and 72 h. EMP, glycolysis; FAB, fructose-diphosphate aldolase; GAPDH, glyceraldehyde 3-phosphate dehydrogenase (phosphorylating); PGK, phosphoglycerate kinase; PGM, phosphoglucomutase; ENO, enolase; PK, pyruvate kinase; PDH, pyruvate dehydrogenase; PPP, pentose phosphate pathway; G6PD, 6-phosphate 1-dehydrogenase; PGD, gluconate 6-phosphate dehydrogenase; PC, pyruvate carboxylase; ME, malic enzyme; TCA, tricarboxylic acid cycle; ICDH, isocitrate dehydrogenase; SCS, succinyl-CoA synthetase; fumC, fumarate hydrolase; MDH, malate dehydrogenase; ACAA, acetyl-CoA acyltransferase; HIBCH, 3-hydroxyisobutyryl-CoA hydrolase; HMGCC, hydroxymethylglutaryl-CoA lyase; BCKDHB, 2-isovalerate dehydrogenase E1 component beta subunit; AUH, methylglutaryl-CoA hydrolase; ACADSB, short-chain 2-methylacyl-CoA dehydrogenase; HADH, 3-hydroxyacyl-CoA dehydrogenase; ilvE, branched-chain amino acid transaminase; BCAAs, branched chain amino acids; FAS2, fatty acid synthase; LACS, long-chain acyl-CoA synthase; ER, enoyl reductase; KR, ketoacyl reductase; KS, ketoacyl synthase; KAR, very long-chain 3-oxoacyl-CoA reductase; HACD, very long-chain (3R)-3-hydroxyacyl-CoA dehydratase; ELO3, elongase 3; ECHS1, enoyl-CoA hydratase; DECR2, 2,4-dienoyl-CoA reductase [(3E)-enoyl-CoA-producing]; ECH1, delta3,5-delta2,4-dienoyl-CoA isomerase; ECH2, peroxisomal enoyl-CoA hydratase 2; SCP2, sterol carrier protein 2; AMACR, alpha-methylacyl-CoA racemase; ACOX1, acyl-CoA oxidase; ACADM, acyl-CoA dehydrogenase; ACAT, acetyl-CoA C-acetyltransferase; ECI2, delta3-delta2-enoyl-CoA isomerase; CYP, NADPH-cytochrome P450 reductase; TGL4, LPA acyltransferase; GK, glycerol kinase; DPPL, diacylglycerol diphosphate phosphatase; PDC, phospholipid:diacylglycerol acyltransferase; MGAT2, 2-acylglycerol O-acyltransferase 2; AGPAT, lysophosphatidate acyltransferase; SOD, superoxide dismutase; CAT, catalase; GSR, glutathione reductase (NADPH); GST, glutathione *S*-transferase. Three biological replicates were carried out.

In oleaginous microorganisms, large quantities of acetyl-CoA are consumed as precursors for the synthesis of fatty acids, which are primarily derived from central carbon metabolism (34). The expression levels of fructose-diphosphate aldolase (FBA), phosphoglucomutase (PGM), enolase (ENO), and pyruvate kinase (PK) involved in the glycolytic pathway were upregulated in the mutant strain at all times compared to the wild type to generate more pyruvate. The pyruvate dehydrogenase genes *pdhB* and *pdhC* are the key enzyme genes that catalyze the production of acetyl-CoA from pyruvate. The analysis showed that the expression of *pdhB* and *pdhC* genes was significantly higher in the mutant than in the wild type at 24, 48, and 72 h. The key enzymes involved in TCA, isocitrate dehydrogenase (ICDH1), succinyl-CoA synthase (SCS), fumarate hydrolase (fumC), and malate dehydrogenase (MDH) were downregulated compared to the wild type during the fermentation cycle. In contrast, the expression of pyruvate carboxylase (PC), which catalyzes the carboxylation of pyruvate to oxaloacetate, and citrate lyase (CS), the rate-limiting enzyme that catalyzes the conversion of oxaloacetate to citrate, was considerably higher in the mutants than in the wild type. Notably, DEG analysis revealed significantly higher (1.24- to 3.39-fold) expression of genes for critical enzymes of the valine, leucine, and isoleucine degradation pathways in the mutant strains than in the wild type at 72 h (Fig. 5). This situation favors the accumulation of acetyl-CoA in the cytoplasm of the mutant (35).

The continuous supply of the reducing agent NADPH is a vital cofactor that is essential for fatty acid synthesis and unsaturation. NADPH is generated primarily through the enzymatic reactions of glucose-6-phosphate 1-dehydrogenase (G6PD), and gluconate 6-phosphate dehydrogenase (PGD) in the pentose phosphate pathway (36). In addition, malic acid undergoes a dehydrogenation reaction catalyzed by malic enzyme (ME) to produce pyruvate and accompanied by NADPH production. During fermentation, the expression levels of all three enzyme genes were higher in the mutant strain than in the wild type, and an adequate pool of NADPH ensured the supply of reducing power required for the synthesis of PUFAs in the mutant strain.

Malonyl-CoA, a direct substrate for fatty acid synthesis, is generated from acetyl-CoA catalyzed by acetyl-CoA carboxylase. This reaction is a key rate-limiting step in fatty acid synthesis (12, 22). Subsequently, the FAS pathway generates PUFAs from saturated fatty acids in the aerobic phase through successive desaturation and extension steps (37). On the other hand, the oxygen-independent PKS pathway has been reported to be the major bearer of PUFAs such as EPA and DHA biosynthesis (38). Malonyl-CoA is catalyzed by a series of key enzymes of the PKS pathway involving condensation by ketoacyl synthase (KS), ketoreduction by ketoacyl reductase (KR), dehydration by dehydratases (DH), and enoylreduction by enoyl reductase (ER) to generate PUFAs. The results were as anticipated; the expression of the acetyl-CoA carboxylase ACACA was consistently considerably higher in the mutant strain than in the wild type (2.39-fold, 2.20-fold, and 4.86-fold at 24 h, 48 h, and 72 h, respectively). This implies that there will be more than sufficient precursors available to flow into the synthetic pathway of PUFAs. The expression of KS, KR, and ER, key enzymes involved in PKS, was differentially upregulated at 24 h, 48 h, and 72 h in the mutant strain compared to the wild type. Apparently, the higher-intensity PKS pathway made an important contribution to the increase in EPA titers in the mutant strain. Interestingly, we observed that fatty acid synthase (FAS2) in the FAS pathway was downregulated during the fermentation cycle, whereas long-chain fatty acid synthesis-related genes, such as long-chain acyl-CoA synthase (LACS), very long-chain (3R)-3-hydroxyacyl-CoA dehydratase (HACD), very long-chain 3-oxoacyl-CoA reductase (KAR), and fatty acid elongase 3 (ELO3), were all expressed at substantially higher levels than the wild type. This result indicates that saturated fatty acids would have a higher chance of being synthesized into PUFAs by extension, which is a reasonable explanation for the much higher percentage of EPA in the fatty acid composition of the mutant than the wild type (as shown in Fig. 2D).

Genes involved in triglyceride biosynthesis, fatty acid degradation, and β-oxidation have been reported to be correlated with lipid metabolism stages (39). Most of the lipids in *Schizochytrium* cells are stored in lipid droplets in the form of triglycerides (TAG) (40). Key genes involved in the triglyceride synthesis pathway were expressed at higher levels with increasing fermentation time in the mutant strain compared to the wild type. Fatty acid β-oxidation leads to fatty acid degradation and altered lipid distribution. Genes related to the fatty acid β-oxidation pathway, enoyl-CoA hydratase (ECHS1), acyl-CoA oxidase (ACOX1), delta3,5-delta2,4-dienoyl-CoA isomerase (ECH1), and sterol carrier protein 2 (SCP2) and genes encoding peroxisomal enoyl-CoA hydratase 2 (ECH2) were significantly less expressed in the mutant than in the wild type. At the same time, the transcription levels of the key enzymes acyl-CoA dehydrogenase (ACADM), delta3-delta2-enoyl-CoA isomerase (ECI2), and NADPH-cytochrome P450 reductase (CYP) genes in the fatty acid degradation pathway in the mutant were considerably downregulated at 48 h. Therefore, the enhancement of triglyceride biosynthesis, the decrease of β-oxidation, and the fatty acid degradation pathway are also significant reasons for the increase of lipid content in mutant DBT-64.

PUFAs are readily oxidized due to their great degree of unsaturation. Lipid peroxidation decreases the PUFA content while leading to the accumulation of intracellular ROS (41). ROS in microalgae principally include superoxide anion, hydrogen peroxide, lipid hydrogen peroxide, and peroxide free radicals (42). Under steady-state physiological conditions, ROS can be scavenged by diverse antioxidant defense components, involving both nonenzymatic and enzymatic mechanisms (43). Superoxide dismutase (SOD) and catalase (CAT) are the main antioxidant enzymes. SOD can catalyze highly toxic superoxide anions to oxygen and hydrogen peroxide (44). Then, CAT catalyzes hydrogen peroxide to generate H_2_O and O_2_ (45). Transcriptomic analysis (Fig. 5) shows that the expression level of CAT in the mutant strain is higher than that in the wild-type strain at three time points, and the expression level of SOD2 in the mutant strain is significantly higher than that in the wild-type strain at 72 h (2.93-fold). This implies that the mutant possesses a higher activity of oxidative stress kinase to resist the intracellular accumulation of ROS. For nonenzymatic defense systems, the mutant encodes glutathione reductase (GSR), and glutathione *S*-transferase (GST) gene expression levels were higher than those of the wild type at three time points. At 48 h, the gene expression levels of GSR and GST in the mutant were 2.73 times and 12.55 times higher than those of the wild type, respectively. Therefore, whether enzymatic mechanism or nonenzymatic mechanism, the oxidative stress system of the mutant was more active, which was beneficial to protect PUFAs from oxidative decomposition and accumulate more PUFAs.

### Verification of gene expression through qPCR.

**FIG 6:**
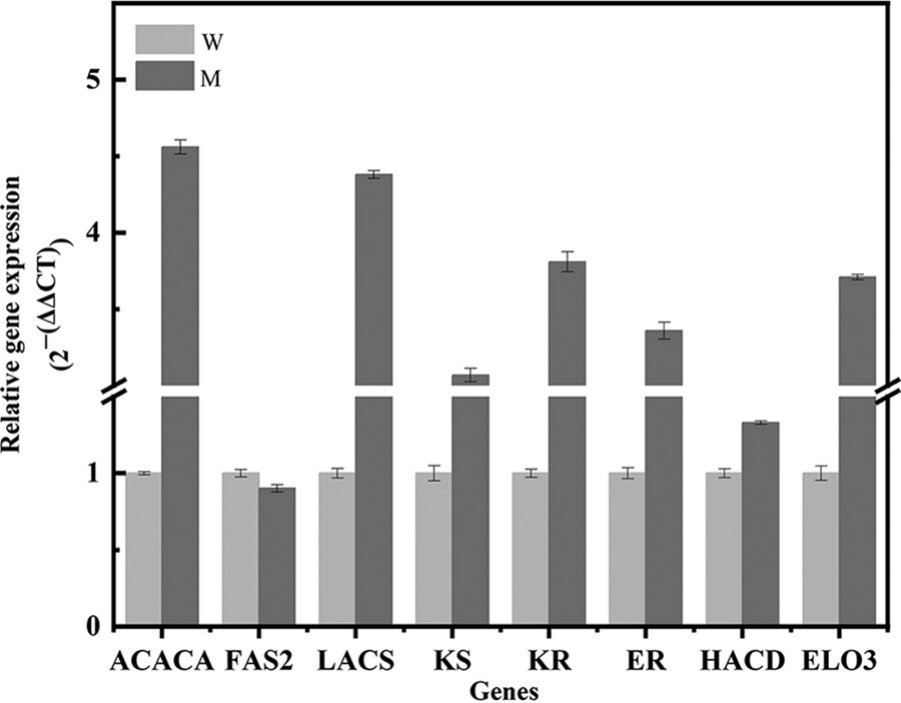
qRT-PCR was used to verify the relative expression levels of related enzyme genes. The error bars represent the standard deviation based on three independent measurements.

We analyzed eight genes correlated with the biosynthesis of fatty acids (ACACA, FAS2, LACS, KS, KR, ER, HACD, and ELO3); these genes were differentially expressed between the wild-type strain and DBT-64 at 72 h. Their expression profiles were verified to validate transcriptome analysis data (Fig. 6). The results demonstrated that the gene expression level acquired by reverse transcriptase quantitative PCR (RT-qPCR) analysis was consistent with that detected by transcriptome sequencing (RNA-Seq) analysis, hence confirming the reliability and stability of RNA-Seq data.

## DISCUSSION

Current research on *Schizochytrium* mainly focuses on improving fatty acid and DHA production using mutagenesis screening methods. In this study, a multiplex screening strategy based on the combination of ARTP and DES mutagenesis was developed, and a mutant strain with high EPA yield was obtained. First, the strain *Schizochytrium* ATCC 20888 was treated with ARTP mutagenesis, and the high-lipid-yielding mutant strain A-32 was obtained using acetyl-CoA carboxylase inhibitor sethoxydim as a screening agent. It was found that the ER structural domain of *Schizochytrium* spp. can play a potential role in PUFA synthesis by catalyzing the reduction of double bonds to EPA through the PKS pathway (46). Meanwhile, the strains with high PUFA content in microorganisms have higher antioxidative stress capacity. Therefore, we used mutant A-32 as the initial strain for DES mutagenesis, and enoyl-ACP reductase inhibitor (triclosan) and ROS inducer (2,2′-bipyridine) were used as compound screening agents to screen high-EPA-yielding mutants. The 4 mutants screened had significantly higher EPA yields and percentages of EPA in total fatty acids. Among them, the mutant DBT-64 had the highest EPA yield, reaching 904.21 mg/L, and the percentage of EPA in the total fatty acid (6.34%) was 4.01 times that of the wild-type strain. As shown in Table 2, the mutants developed in this study obtained the highest EPA production in shake flask culture. At the same time, the production performance of DBT-64 in the fed-batch fermentation process is second only to that described in Wang et al. (47), but it still has an advantage in the proportion of EPA. In a recent study, Ma et al. achieved an EPA yield of 2.25 g/L through a cobalamin-deficient fermentation process (48), which may provide a new idea for our future work.

**TABLE 2:** Comparative results of EPA yield and percentage among different studies

Strain	Exptl conditions	Strategy	EPA percentage (% of total FAs)	EPA yield (mg/L)	Reference or source
*Schizochytrium* sp. strain MYA1381	Fed-batch fermentation	Overexpressed the malonyl-CoA:ACP transacylase domain	1.45	1,650.00	12
*Schizochytrium* sp. strain HX-308	Shake-flask fermentation	Replaced the AT domain	3.84		52
*Schizochytrium* sp. strain MYA1381	Shake-flask fermentation	Addition of 50 mg/L fluoridone	0.65	151.00	9
Schizochytrium limacinum SR21	Fed-batch fermentation	Replacement of the enoyl reductase gene	1.00	732.40	53
*Aurantiochytrium* sp. strain SD116	Fed-batch fermentation	Expression of the EPA synthetic gene cluster	3.05	2,700.00	47
*Schizochytrium* sp. strain DBT-64	Shake-flask fermentation	ARTP-DES mutagenesis	6.34	904.21	This study

Transcriptome analysis showed that under nutrient-sufficient conditions, the expression of key genes in the glycolytic pathway was upregulated in the mutant strain, while the expression of essential genes in the TCA cycle was downregulated in the mutant strain, a situation that may facilitate the synthesis of more acetyl-CoA in the cytoplasm. Enhanced valine, leucine, and isoleucine degradation pathways could accumulate more acetyl-CoA, while the expression level of acetyl-CoA carboxylase is significantly increased to provide a large amount of malonyl-CoA for fatty acid synthesis. Upregulation of ME in the mutant strain and G6PD and PGD expression in the pentose phosphate pathway (PPP) provided more NADPH for fatty acid synthesis. Existing theories suggest that *Schizochytrium* spp. may synthesize fatty acids through the FAS and PKS pathways (9, 49). Compared to wild-type strains, the FAS gene in the mutant was downregulated during the fermentation stage, but the crucial genes KS, KR, and ER in the PKS pathway were significantly upregulated. Also, the expression levels of genes involved in long-chain synthesis were also significantly upregulated compared with the wild type. Analysis of the fatty acid composition of the mutant strain and the wild type revealed (Fig. 2D) that the percentage of EPA in the total fatty acids of the mutant strain was much higher than that of the wild type, but the percentage of DHA was lower than that of the wild type by 11.96%. Therefore, it is reasonable to speculate that the biosynthesis of EPA and DHA occur in different branches and compete for common substrates. Hayashi et al. also proposed that EPA and DHA may be synthesized through different pathways (50), which is consistent with our idea. Another possibility is that the biosynthesis of EPA and DHA proceeds through the same pathway, and EPA is upstream of the pathway, and the expression intensity of the important enzymes involved in catalyzing the synthesis of EPA to DHA decreases, leading to an increase in the yield of EPA and a decrease in the flux of DHA synthesis.

### Conclusions.

In this study, a new mutagenesis strategy based on ARTP-DES compound mutagenesis combined with multiple screening pressures was proposed to obtain mutants with high EPA yields in *Schizochytrium*. After fed-batch fermentation, the mutant strain DBT-64 had 1.45 times higher lipid content than the wild type, reaching 59.17% (percent biomass) and an EPA titer of 1.86 g/L. The transcriptome results showed that the upregulation of genes related to the PKS pathway, fatty acid elongation, and the triglyceride synthesis pathway in the mutant strain and those related to the downregulation of the expression levels of genes involved in the fatty acid degradation pathway may favor the biosynthesis of EPA in *Schizochytrium*. This study provides an efficient method for the selection and breeding of high-EPA-producing *Schizochytrium* spp. and valuable information for regulating the proportion of EPA in microalgal oil by means of genetic engineering.

## MATERIALS AND METHODS

### Strains and chemicals.

*Schizochytrium* ATCC 20888 was purchased from the American Type Culture Collection (ATCC). Fatty acid standard was purchased from Nu-Chek Prep, Inc. (Elysian, MN, USA). Triclosan and 2,2-bipyridine were purchased from Sangon Biotech Co., Ltd. (Shanghai, China). Sethoxydim and diethyl sulfate were purchased from ANPEL Laboratory Technologies Co., Ltd. (Shanghai, China). All other chemicals were of analytical grade.

### Media and culture conditions.

The strain preserved in a glycerin tube was inoculated into a 250-mL shake flask containing 50 mL seed culture medium and cultivated at 28°C and 200 rpm for 48 h. After two generations of cultivation, seed culture (10%, vol/vol) was inoculated into a 500-mL shake flask containing 100 mL fermentation medium and cultivated at 28°C and 200 rpm for 108 h.

The composition of basal, seed, and fermentation medium followed the previous study (51). The basal medium comprises 5 g/L glucose, 1 g/L peptone, 1 g/L yeast extract, and 17.5 g/L sea salt. The seed medium contains 30 g/L glucose, 10 g/L peptone, 5 g/L yeast extract, 0.05 g/L vitamin B1, 0.05 g/L vitamin B6, 0.0005 g/L vitamin B12, and 15 g/L sea salt. The fermentation medium contains 100 g/L glucose, 5.6 g/L peptone, 20 g/L C_5_H_8_NO_4_Na-H_2_O, 2.5 g/L KH_2_PO_4_, 7.2 g/L MgSO_4_, 12.8 g/L Na_2_SO_4_, 0.4 g/L CaCl_2_, 0.1 g/L vitamin B_1_, 0.1 g/L vitamin B_6_, 0.001 g/L vitamin B_12_, and 17.5 g/L sea salt.

The fed-batch fermentation was carried out in a 7.5-L magnetically stirred glass fermenter, and the seed liquid in the logarithmic growth phase was inoculated into 3 L of the initial fermentation medium at an inoculation amount of 10% (vol/vol). The fermentation conditions were 28°C, pH 6.5, stirring speed of 400 rpm, and ventilation volume of 2 VVM. VVM (air volume/culture volume/min) refers to the ratio of the ventilation rate per minute to the actual liquid volume of the tank. The pH was controlled at 6.5 by adding 2 M NaOH or 2 M HCl. During the fed-batch fermentation process, 30 mL of fermentation broth was taken every 12 h until the end of the fermentation, cells were collected, and the dry biomass, lipid, and PUFA yield were measured. When the glucose concentration in the fermentation broth was less than 20 g/L, add 600 g/L of glucose solution was added until the glucose concentration in the fermentation broth was about 50 g/L.

### Mutagenesis and screening.

Pipettes (100 μL) of the exponential-phase bacterial suspension were spread on solid plates containing 0, 10, 20, 30, 40, 50, and 60 mg/L sethoxydim, and the plates cultivated at 28°C for 4 days. Three replicate plates were set up for each concentration of screening agent. Individual colonies on the plates were manually counted and used equation 1 to determine lethality (%):

(1) $$\text{Lethality rate}\;\left(\%\right)=\frac{\text{control colonies }-\text{ survival colonies}}{\text{control colonies}}\times 100\%$$

The original strain was inoculated into the seed medium and cultivated for 48 h to obtain the exponential-phase seed solution. Then, the cell pellet was collected by centrifugation and washed with 0.2 M sterile phosphate-buffered saline (PBS) buffer (pH 7.2). The OD_595_ value of the seed solution was adjusted to 0.6 to 0.7. ARTP mutagenesis was performed using the ARTP breeding system (ARTP-III, Siqingyuan Bio-technology Co., Ltd., China). Seed culture suspension (10 μL) was spread evenly on a sterile ARTP-specific metal carrier and then the metal carrier was put into an ARTP processing chamber with a helium flow rate of 10 L/min and a power of 100 W, and the processing times were 0, 10, 20, 30, 40, 50, and 60 s. After mutagenesis, the metal supports were eluted with sterile PBS buffer (pH 7.2), and the cells were eluted into sterile centrifuge tubes. The resulting cell suspension was appropriately diluted, and 100 μL was pipetted onto basal medium plates and incubated at 28°C for 4 days. The ARTP treatment time was optimized by lethality measurement. Mutants were screened on basal medium plates supplemented with 40 mg/L sethoxydim. Colonies were manually counted, and the lethality (%) of the ARTP treatment was calculated using equation 1.

**FIG 7:**
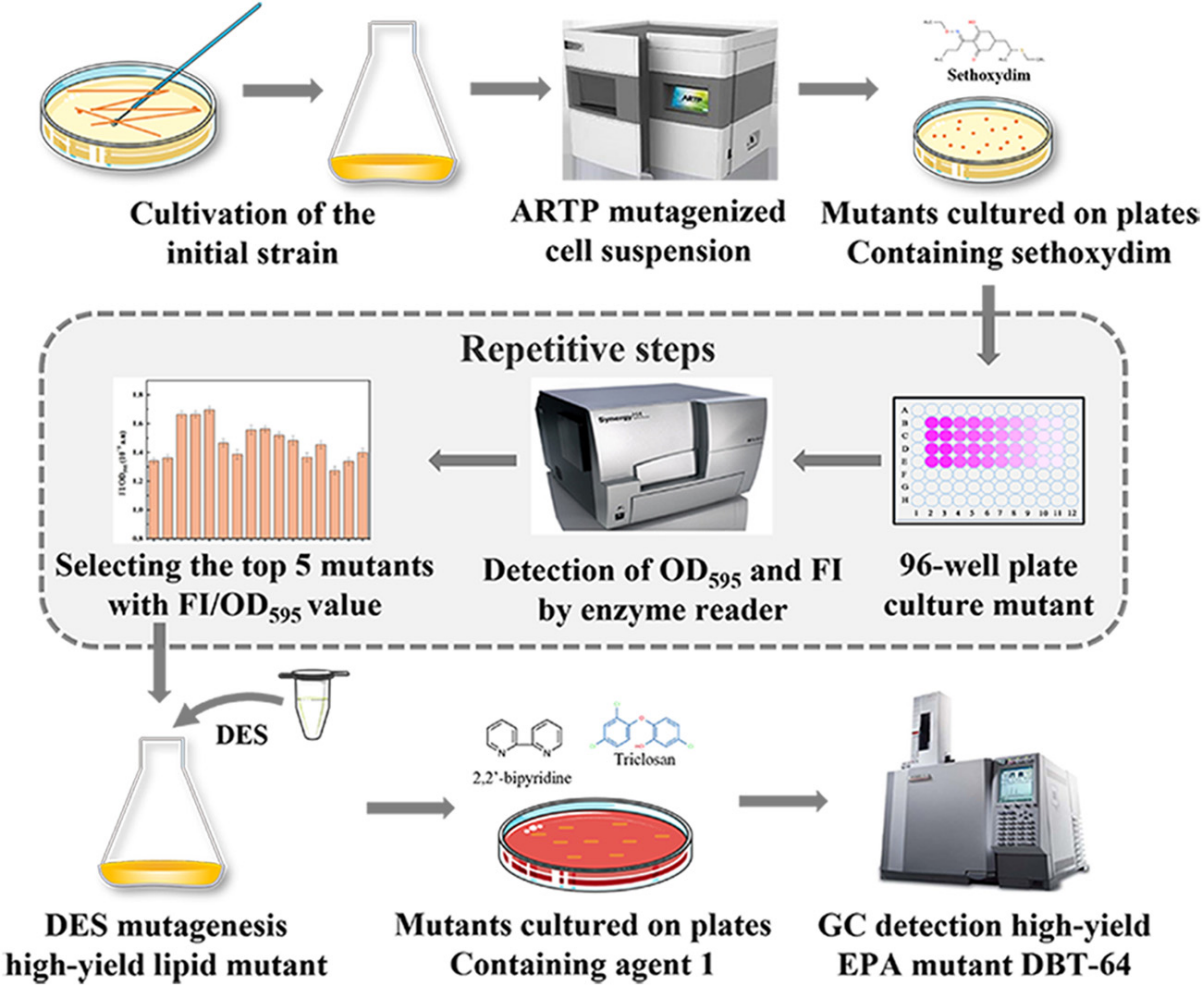
ARTP-DES compound mutagenesis program and rational screening of multiple pressures (sethoxydim, triclosan-2,2′-bipyridine).

The mutants with larger plates were selected and inoculated into a 48-well cell culture plate containing 1 mL of fermentation medium and cultured for 4 days. Culture solution (200 μL) was placed in a 96-well transparent plate. The OD_595_ value was measured with a microplate reader, and then the culture solution was transferred to a black 96-well transparent-bottom plate; and 50 μL of 20% dimethyl sulfoxide (DMSO) and 5 μL of 100 μg/mL Nile Red staining solution were added, and after incubation at 25°C for 20 min in the dark, fluorescence was detected with a 485/20-nm excitation filter and a 595/20-nm emission filter. The top five mutants with the FI/OD_595_ values in each of the two plates were screened, repeated screening was performed, and the plates were transferred for storage. Mutants with high lipid content were selected as starting strains for the second round of diethyl sulfate (final concentration, 1% [vol/vol]) mutagenesis and screening based on triclosan (3.75 mg/L) and 2,2′-bipyridine (6.25 mg/L) complex pressure. The mutagenesis and screening procedures are shown in Fig. 7.

### Detection of microalgal biomass, lipid, EPA, and fatty acids.

Fermentation broth (30 mL) was centrifuged at 8,000 rpm for 15 min, washed twice with distilled water, and freeze-dried *in vacuo*, and the biomass was determined gravimetrically. The glucose concentration in the fermentation broth was measured using a bioanalyzer (SBA-40C, Institute of Biology, Shandong Academy of Sciences). Lipids were extracted and fatty acid content was measured according to the described method (51).

### Preparation for laser scanning confocal microscopic (LSCM) observation.

First, the culture was diluted to 10^5^ cells/mL, and then 1.5 mL of the diluted solution was added to 250 μL of dimethyl sulfoxide and 15 μL of 0.75 g/mL Nile Red solution stain at 37°C for 30 min in the dark; the mixture was then placed in an LSCM device (C2 confocal microscope system, Nikon, Japan). Using argon laser excitation at 488 nm, the Nile Red fluorescence of the labeled oil droplet morphology was detected in the 560- to 600-nm filter channel to acquire differential interference contrast and fluorescence images.

### RNA isolation, library construction, and sequencing.

For transcriptome analysis, mutant DBT-64 (M) and the wild-type strain (W) were cultured for 24 h, 48 h, and 72 h. A sample of the fermentation broth was collected, and it was centrifuge (5,000 rpm, 5 min) with biological repeats three times. Cells were collected and instantly frozen with liquid nitrogen. Then, total RNA was isolated by adding TRIzol reagent (Invitrogen Life Technology, USA) for transcriptome information analysis. The mRNA was randomly cleaved into small fragments of roughly 300 bp in break buffer by divalent cations at high temperature for cDNA synthesis. The sequencing library was sequenced on a NovaSeq 6000 platform (Illumina).

### Annotation functional genes and identification of differentially expressed genes.

By eliminating the reads of the connector, low-quality bases, and reads with unknown base N content greater than 5%, the original reads were filtered to obtain high-quality reads. The expression level of each gene was standardized by FPKM to compare the fold changes between samples (log_2_ FC). For screening of differentially expressed genes (DEGs) between the wild-type strain and the high-EPA-yielding mutant strain DBT-64, genes with a *P* value of <0.05 and log_2_ fold change absolute value of ≥1 were considered differentially expressed. For functional enrichment, such as KEGG, the differentially expressed genes (DEGs) were mapped to terms in the KEGG database (http://www.genome.jp/kegg). Cluster Profiler software was used to carry out the enrichment analysis of the KEGG pathway of differential genes, focusing on the substantial enrichment pathway with a *P* value of <0.05.

### Quantitative PCR (qPCR) validation.

The qPCR primers (Table S1) were designed utilizing Primer Premier 5.0 software and synthesized by Sangon Biotech (Shanghai, China). cDNA was obtained using Maxima reverse transcriptase (Thermo Scientific, USA). The QuantStudio 1 Plus RT-qPCR system (Thermo Fisher Company) and 2× SG fast qPCR master mix (High Rox) (BBI, USA) were used for quantitative PCR, and 18s rDNA was used as the internal standard. The results were analyzed with the 2^−ΔΔ^*^CT^* method. Three technical repeats were carried out for each sample.

### Data availability.

The raw RNA-Seq data are available at the NCBI Sequence Read Archive (SRA) under BioProject accession no. PRJNA921706.
