# Supplementary material for: Transcriptome Analysis Reveals an Eicosapentaenoic Acid Accumulation Mechanism in a Schizochytrium sp. Mutant
Source: Microbiol Spectr. 2023 Apr 24;11(3):e00130-23. doi: 10.1128/spectrum.00130-23 (PMC10269799; doi:10.1128/spectrum.00130-23)
Supplement: Supplemental file 1 — Supplemental material. Download spectrum.00130-23-s0001.pdf, PDF file, 0.5 MB [file spectrum.00130-23-s0001.pdf]

Transcriptome Analysis Reveals EPA Accumulation Mechanism in *Schizochytrium* sp. Mutant

Ying Ou[a], Yaqi Li[a], Shoushuai Feng[a], Qiong Wang[b,*], Hailin Yang[a,*]

[a] The Key Laboratory of Industrial Biotechnology, Ministry of Education, School of Biotechnology, Jiangnan University, 1800# Lihu Road, WuXi 214122, People's Republic of China

[b] Department of Clinical Laboratory, The Affiliated Wuxi People's Hospital of Nanjing Medical University

*Correspondence authors

Email: wangqiong_john@163.com (Qiong Wang), bioprocessor@126.com (Hailin Yang)

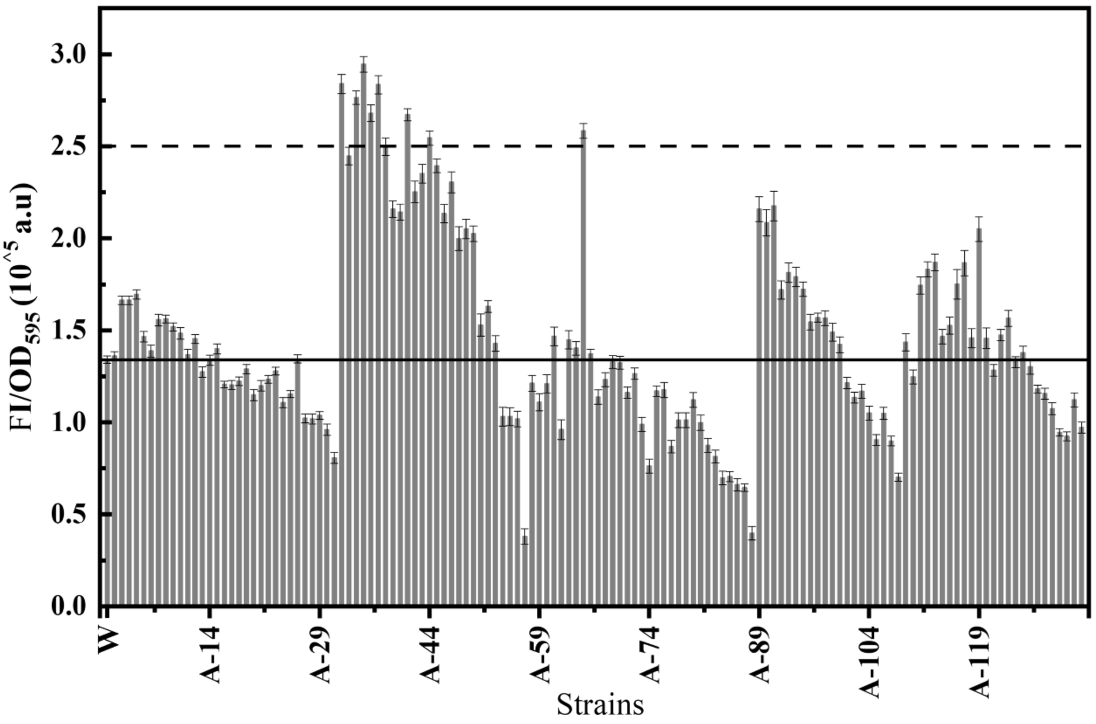

Fig.S1 FI/OD$_{595}$ value of mutants isolated after ARTP mutagenesis and screening sethoxydim. "W" means wild type strain *Schizochytrium* ATCC20888. The solid line indicates FI/OD$_{595}$ of wild type strain, and the dotted line indicates the position where FI/OD$_{595}$ reaches 2.5 *10 $^{\wedge 5}$. The error bars represent the standard deviation based on three independent measurements.

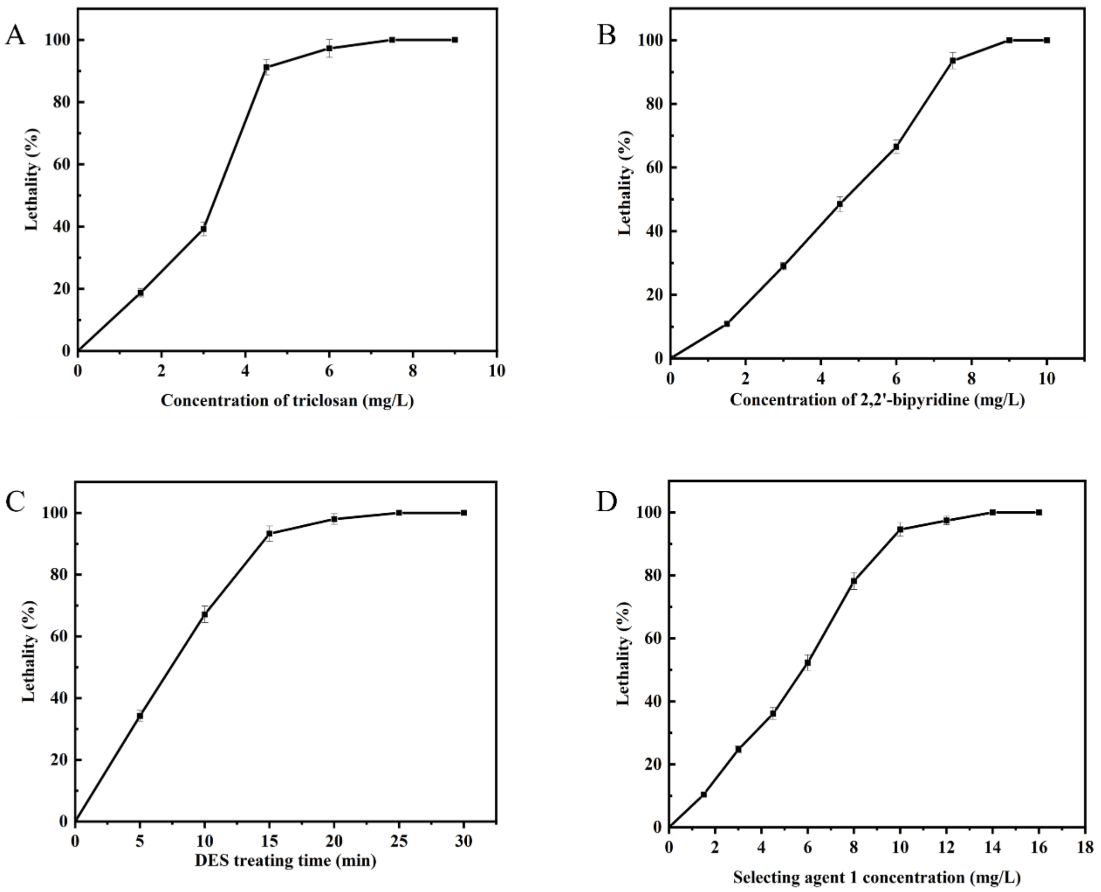

Fig.S2 Lethality of *Schizochytrium* A-32 by DBT mutagenesis and selected agent 1. (A) triclosan; (B) 2,2'-bipyridine; (C) DES treating time; (D) selected agent 1(the ratio of triclosan and 2,2'-bipyridine was 3:5). The error bars represent the standard deviation based on three independent measurements.

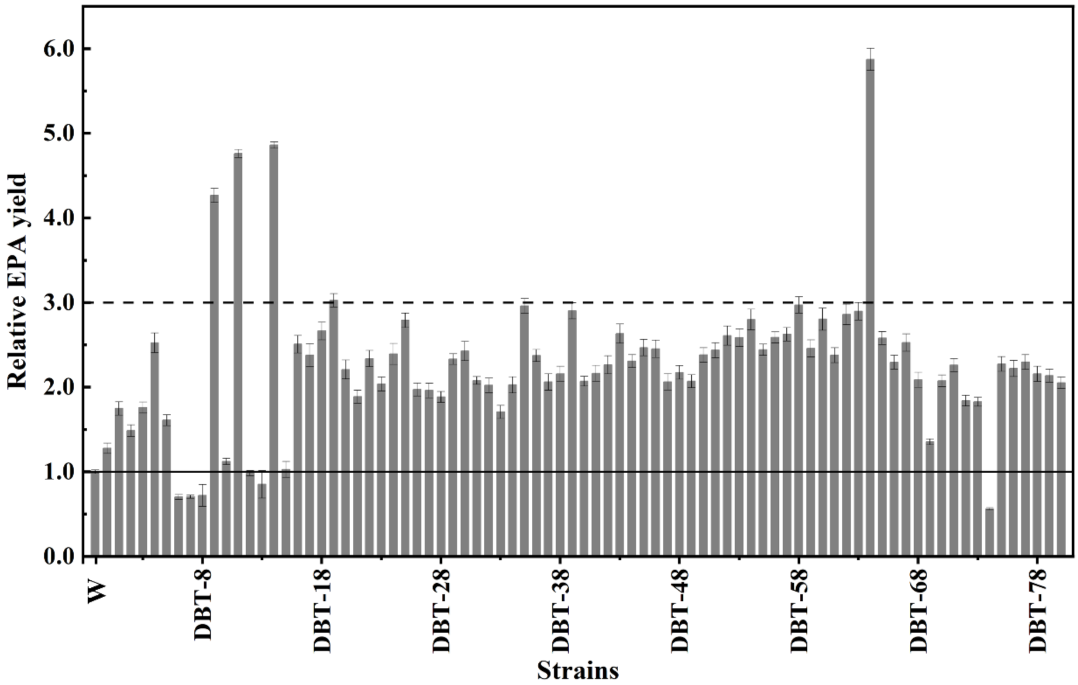

Fig.S3 The relative EPA yield of the isolated mutants after DES mutagenesis and screening agent 1 (triclosan-2,2'-bipyridine). The "w" indicated the wild type strain *Schizochytrium* ATCC20888, and its EPA yield was set as 1.0. The solid line was for the wild type strain, and dotted line indicated the position where the EPA yield increased by 2-fold compared with the wild strain. The error bars represent the standard deviation based on three independent measurements.

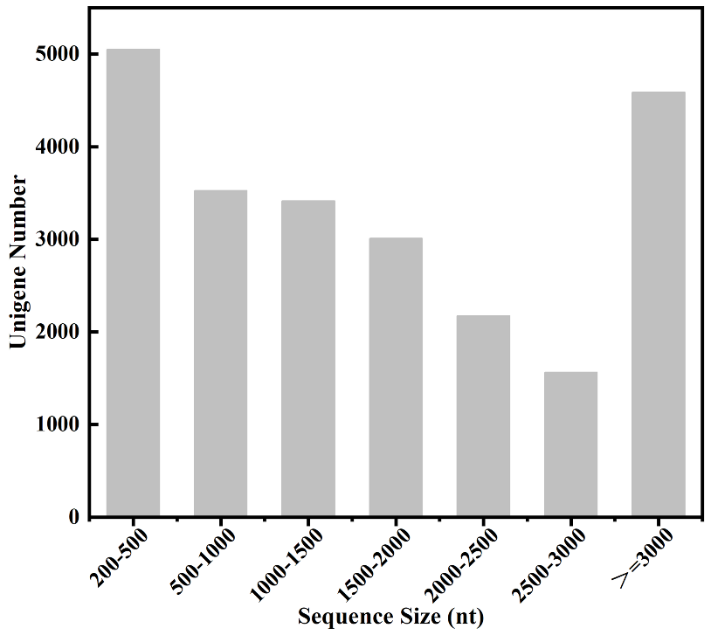

Fig.S4 Length distribution of assembled transcripts. Three biological replicates were carried out.

Table S1 Primer sequence and amplification length of related genes in qPCR.

Gene name	Forward primer (5'-3')	Reverse primer (5'-3')	Amplification length (bp)
18S	GGGTGACGGGGAGTTAGG	CTCATTCCAATAGCAAGACGC	161
FAS2	GTAGTCGCCAAAGGACGG	TACGGCGATGCCGAGG	105
LACS	ACACGGCGCTGAGTGGCG	ACGCCGGCGCGAAGC	148
KS	CTCCCTCACTTGCCTCGCGC	TGGGAGCACCACGCTTCCG	117
KR	AAGGTCTGTGCGCGGCG	GCCGGCGTCATCGGAAAGC	172
HACD	GGAGTACGCGGTTTCGGGCG	CCAGGCCGTCGCGTGC	155
ELO3	GATGGCTCCCCCCTG	CCAGAGAACGAGCAGAGG	147
ACACA	GGGCATCAAGACCACG	AGCGCGAGACAATGTC	194
ER	GACCTTGACCCCGCAGCTC	GGGTGATCTCGCCGGACTTCA	182

Table S2 The biomass, lipid and EPA contents of mutant strain *Schizochytrium* DBT-64 during the twenty-generations subculture.

Generation	Biomass (g/L)	Lipid content (%biomass)	EPA (%TFAs)	EPA yield (mg/L)
W	24.12±0.81	40.09±1.96	1.58±0.07	153.17±16.13
DBT-64-1nd	24.56±1.03	58.07±2.80	6.34±0.22	904.21±35.27
DBT-64-5th	25.14±1.16	57.29±2.27	6.26±0.16	901.61±30.31
DBT-64-10th	24.98±0.95	57.85±2.49	6.24±0.24	901.74±29.78
DBT-64-20th	24.43±0.98	58.11±2.50	6.36±0.27	902.86±18.54

Table S3 Summary of transcriptome sequencing for wild type *Schizochytrium* sp. ATCC20888 and mutant.

Samples	Total Raw	Total Clean		Clean Reads		
	Reads (Mb)	Reads (Mb)	Bases (Gb)	Q20(%)	Q30(%)	Ratio (%)
M24-1	43.69	42.63	6.39	97.31	92.10	97.56
M24-2	43.69	42.41	6.36	97.32	92.16	97.08
M24-3	43.69	42.50	6.37	97.16	91.72	97.27
M48-1	43.69	42.60	6.39	96.95	91.17	97.49
M48-2	43.69	42.39	6.36	97.08	91.51	97.03
M48-3	45.44	43.42	6.51	97.28	91.84	95.55
M72-1	43.69	42.5	6.38	97.09	91.33	97.28
M72-2	43.69	42.63	6.39	97.05	91.17	97.58
M72-3	43.69	42.61	6.39	97.20	91.60	97.53
W24-1	45.44	43.38	6.51	97.22	91.06	95.46
W24-2	43.69	42.44	6.37	97.00	91.29	97.15
W24-3	43.69	42.55	6.38	96.84	90.89	97.39
W48-1	43.69	42.32	6.35	97.11	91.65	96.87
W48-2	43.69	42.25	6.34	96.83	90.84	96.7
W48-3	43.69	42.38	6.36	96.89	90.98	97.00
W72-1	43.69	42.36	6.35	96.94	91.19	96.96
W72-2	43.69	42.53	6.38	97.11	91.58	97.34
W72-3	43.69	42.33	6.35	96.97	91.22	96.88

Q20: the rate of bases in which quality was greater than 20 in clean reads; Q30: the rate of bases in which quality was greater than 30 in clean reads.

Table S4 Quality metrics of transcriptome and unigenes assembly for wild type *Schizochytrium* sp. ATCC20888 and mutant.

Samples	Total Number	Total Length	Mean Length	N50	N70	N90	GC (%)
M24-1	16035	22933891	1430	2072	1469	760	57.46
M24-2	16190	22867935	1412	2049	1461	741	57.46
M24-3	15922	22890727	1437	2067	1475	759	57.50
M48-1	15451	23316968	1509	2149	1534	817	57.61
M48-2	15661	23445472	1497	2148	1521	806	57.67
M48-3	16384	23914158	1459	2117	1487	774	57.64
M72-1	15568	22656264	1455	2072	1478	788	57.75
M72-2	15939	23208049	1456	2085	1490	782	57.77
M72-3	15677	22977775	1465	2089	1493	793	57.73
W24-1	15247	22991692	1507	2161	1529	804	57.85
W24-2	18676	24888067	1332	1995	1387	644	56.38
W24-3	15298	23024503	1505	2150	1516	805	57.88
W48-1	15780	23803970	1508	2161	1536	803	57.86
W48-2	15521	23547741	1517	2159	1532	816	57.94
W48-3	15496	23618196	1524	2159	1530	818	57.92
W72-1	15474	23920341	1545	2208	1554	837	57.77
W72-2	15590	23742013	1522	2180	1540	813	57.82
W72-3	15496	24010436	1549	2212	1563	835	57.74
All-Unigene	23326	44907986	1952	2956	1976	1044	56.97

N50: 50% of the total length is contained in transcripts that are equal to or larger than this value; N70: 70% of the total length is contained in transcripts that are equal to or larger than this value; N90: 90% of the total length is contained in transcripts that are equal to or larger than this value; GC (%): the percentage of G and C bases.
